# Background incidence rates from electronic healthcare databases for vaccine safety monitoring: review of challenges from the COVID-19 vaccination campaign and proposal for best practices

**DOI:** 10.3389/fdsfr.2025.1651090

**Published:** 2025-12-01

**Authors:** Sonja Gandhi-Banga, Laurence Serradell, Deborah Layton, Jill Dreyfus, Nicolas Praet, Diana C. Garofalo, Vincent Bauchau, Scott P. Kelly, Lin Li

**Affiliations:** 1 Epidemiology and Benefit Risk, Patient Safety and Pharmacovigilance, Sanofi, Toronto, ON, Canada; 2 Epidemiology and Benefit Risk, Patient Safety and Pharmacovigilance, Sanofi, Lyon, France; 3 PEPI Consultancy Limited, Southampton, United Kingdom; 4 Global Medical Epidemiology, Pfizer, Inc, New York, NY, United States; 5 Global Epidemiology Organization, Johnson and Johnson, Beerse, Belgium; 6 Independent, Previously Vaccine Safety, GlaxoSmithKline (GSK), Wavre, Belgium; 7 Epidemiology and Benefit Risk, Patient Safety and Pharmacovigilance, Sanofi, Morristown, NJ, United States

**Keywords:** adverse events of special interest, background incidence rates, best practices, electronic healthcare databases, safety monitoring, COVID-19 vaccine

## Abstract

Background incidence rates (BIRs) are essential for contextualizing adverse event rates in vaccine safety monitoring, particularly through observed-to-expected (O/E) analyses. The unprecedented rapid development of vaccines during the coronavirus disease 2019 (COVID-19) pandemic necessitated rigorous and continuous safety monitoring. While BIRs are traditionally obtained from literature reviews, the pandemic accelerated the large-scale generation of BIRs from electronic healthcare databases through various initiatives such as the vACCine covid-19 monitoring readinESS (ACCESS) and the Biologics Effectiveness and Safety (BEST) to support COVID-19 vaccine safety surveillance strategies. The Beyond COVID-19 Monitoring Excellence (BeCOME) initiative, launched in 2022, established seven working groups, including one focused on best practices for BIR generation and utilization in pharmacovigilance activities. The BeCOME BIR working group conducted a targeted literature review to enable focused analysis of challenges that emerged during large-scale vaccination campaigns through December 2023. The group also used structured group discussion following the nominal group technique over 10 months to develop consensus-based judgments on critical factors for BIR best practices. The findings were organized into four domains: key initiatives summary, pandemic-related challenges, implications for O/E analyses, and best practice recommendations. To identify key BIR initiatives supporting COVID-19 vaccine safety assessment, the review employed multiple strategies including scientific literature examination, public health authority website reviews, and reference list searches; data on study characteristics, limitations, challenges, and recommendations were extracted. The targeted review focused on five major initiatives, such as ACCESS and BEST, that generated BIRs for adverse events of special interest (AESIs) during the pandemic. The group identified persistent challenges during the vaccination campaign including timeliness constraints during rapid vaccine deployment, substantial heterogeneity across data sources, inconsistent case definitions, limited information for key subpopulations, and difficulties addressing emerging AESIs. These challenges directly impacted O/E analyses, potentially leading to biased safety signal assessments. We propose a coordinated action plan among key stakeholders to establish sustainable mechanisms for regular BIR delivery with methodological improvements, develop consensus on best practices for BIR selection, and secure resources to ensure pandemic preparedness. Implementing these recommendations will strengthen vaccine safety monitoring systems for both routine vaccination programs and future public health emergencies.

## Introduction

Background incidence rates (BIRs) of potential adverse events of special interest (AESIs) - rates at which these outcomes naturally occur in the general or specific populations of interest - are important for contextualizing the rates observed in populations exposed to vaccines or drugs of interest (Black et al., 2021). BIR data play a key role in safety monitoring across various settings, including clinical trials, observational real-world evidence (RWE) studies, and spontaneous reporting systems. They are also central to conducting advanced quantitative analyses, such as observed-to-expected (O/E) analyses. In the context of vaccines, O/E analyses compare the observed number of adverse events in a vaccinated population (typically obtained from a spontaneous reporting system) to the expected number calculated using BIRs from general or specific populations (comparator population) to assess safety signals (Mahaux et al., 2016).

After the World Health Organization (WHO) declared the coronavirus disease 19 (COVID-19) outbreak caused by the severe acute respiratory syndrome coronavirus 2 (SARS-CoV-2) a global pandemic on 11 March 2020 (World Health Organization, 2025), COVID-19 vaccines were developed at an unprecedented speed and were made available for mass vaccination campaigns within a year (Ledford et al., 2020; Fortner and Schumacher, 2021). While the rapid development of vaccines was a crucial tool for controlling the pandemic, it also underscored the need for rigorous and ongoing safety monitoring. As with all medicinal products, monitoring the safety of COVID-19 vaccines in real-world settings, in addition to data generated through clinical trials, was (and still is) a key component of pharmacovigilance (PV). Real-world monitoring is particularly important since rare adverse events not detected/detectable during the clinical development phases could emerge during large-scale vaccination campaigns.

Public health or national health institutions, regulatory agencies, academic institutions, healthcare organizations, and vaccine manufacturers proactively developed vaccine safety surveillance strategies in anticipation of the COVID-19 vaccine rollout during the pandemic. Lists of AESIs for COVID-19 vaccines were issued by multiple organizations, including but not limited to the Safety Platform for Emergency Vaccines (SPEAC), the US Centers for Disease Control and Prevention (CDC), the European Medicines Agency (EMA), the US Food and Drug Administration (FDA), and the Medicines and Healthcare products Regulatory Agency (MHRA) in the UK (Black et al., 2021). These lists were extensive, covering multiple systems—neurologic, immunologic, cardiologic, hematologic, among others—due to the emerging nature of the COVID-19 disease, and the use of different and/or novel vaccine platforms. They were also dynamic and not necessarily consistent, with some AESIs being included in certain lists but not in others (Black et al., 2021). In addition, there was no single definition of AESIs. For example, the WHO defines an AESI in the context of COVID-19 as “a pre-specified medically-significant event that has the potential to be causally associated with a vaccine product that needs to be carefully monitored and confirmed by further special studies” with an emphasis of specificity (World Health Organization, 2020). In contrast, the Council for International Organizations of Medical Sciences (CIOMS) adopts a more sensitivity-focused approach, defining an AESI as a noteworthy event related to a specific product or class of products that a sponsor wants to monitor carefully. These events could be either serious or non-serious and might include potential precursors or prodromes for more serious medical condition (Council for International Organizations of Medical Sciences CIOMS, 2023). The heterogeneity of AESI lists might have reflected different interpretations of AESI definitions across these organizations during the pandemic, thus affecting operational implementation across regulatory and public health settings.

Prior to the COVID-19 pandemic, BIRs for given AESIs were typically obtained from literature reviews of published population-based epidemiological studies or generated from retrospective analyses of electronic healthcare databases (Black et al., 2021; Mahaux et al., 2016; Esposito et al., 2018; Gubernot et al., 2021; Klein et al., 2010; Willame et al., 2021a). However, this approach is constrained by inconsistent design or methodological choices across studies and reliance on historical data might not have reflected current population health patterns or emerging conditions. The urgency of the COVID-19 pandemic drove a move toward large-scale generation of BIRs from electronic healthcare databases, enabling rapid evaluation of potential safety signals from the extensive and evolving lists of AESIs. To prepare for the aforementioned safety surveillance strategies for COVID-19 vaccines, various initiatives were launched over the course of the pandemic to generate BIRs for many overlapping AESIs from electronic healthcare databases to support analyses by health authorities and marketing authorization holders (MAHs) in line with regulatory requirements for systematic O/E analyses in their risk management plans. In Europe, the EMA funded the vACCine covid-19 monitoring readinESS (ACCESS) project, which generated rates for 41 AESIs in 10 European healthcare databases (Willame et al., 2023; Willame et al., 2021b). In the US, the FDA Biologics Effectiveness and Safety (BEST) initiative generated BIRs for 17 AESIs in six US administrative claims databases (Moll et al., 2023; Tworkoski et al., 2021). In addition, the Observational Health Data Sciences and Informatics (OHDSI) community generated and published rates of 15 AESIs in eight countries in 2021 (Li et al., 2021), and rates of 16 AESIs in eleven countries in 2023 (Voss et al., 2023). The Global COVID Vaccine Safety (GCoVS) project was established in 2021 under the multinational Global Vaccine Data Network (GVDN) consortium with the aim to gather rates on 13 AESIs in 9 countries (Phillips et al., 2023). Other groups from Canada, Korea, Scotland, and Australia also published BIRs of prespecified AESIs (Nasreen et al., 2021; Nasreen et al., 2022; Huh et al., 2021; Cullen et al., 2023; Pillsbury et al., 2023). The abundance of newly available BIR data combined with BIRs from existing literature facilitated the ability to conduct O/E analyses while also presenting challenges in utilizing and interpreting the results.

Beyond COVID-19 Monitoring Excellence (BeCOME) was launched in 2022 to identify lessons learned from non-competitive cooperation among vaccine manufacturers during the COVID-19 pandemic regarding the real-world monitoring of benefits and risks of vaccines (Bauchau et al., 2023; Bauchau et al., 2025). Seven working groups were established, with one dedicated to identifying common challenges, possible solutions, and priorities across stakeholders for sustainable BIR generation from electronic healthcare databases and its utilization. Specific objectives of this BIR working group were to (i) identify and summarize information on published initiatives that were conducted to generate BIRs for COVID-19 vaccine safety monitoring, (ii) review challenges encountered in generating and using BIRs in the framework of PV activities, and (iii) propose best practices for BIR generation and use. This paper summarizes the working group’s findings in relation to these objectives and offers a set of broad recommendations intended to cover the PV scope of any vaccine, both within and outside the context of pandemic preparedness, drawing from the experience gained during the COVID-19 pandemic.

## Methods

### Working group structure and experience

The manuscript was prepared by nine volunteer members of the BeCOME BIR working group, who are current or former PV and pharmacoepidemiology experts of various research affiliations (pharmaceutical companies, contract research organizations and independent consultancy) based in Canada, Europe, UK and US with specific experience in COVID-19 vaccine safety surveillance and/or safety studies. While this manuscript describes MAH experiences and lessons learned, objectivity is maintained through the use of published evidence, systematic consensus development, and integration of diverse stakeholder perspectives (see targeted literature review and consensus approach below for methodological details).

### Identification of published initiatives on BIRs through targeted literature review

Rather than conducting a systematic review, a targeted literature review approach was selected and conducted by working group members to identify and synthesize key initiatives that generated BIRs to support the assessment of COVID-19 vaccine safety by the MAHs during the WHO’s Public Health Emergency of International Concern (ending on 5 May 2023) (World Health Organization, 2025). The search covered publications and reports available through 31 December 2023. This approach enabled a focused analysis and review of the challenges encountered by the MAHs during post-marketing monitoring, with particular emphasis on issues that emerged during large-scale vaccination campaigns. To this end, the working group used multiple strategies including (i) a targeted search and review of the scientific literature that were known, available and actively used by the MAHs during this specific period (ii) a targeted search and review of prespecified Public Health and Health Authority websites such as EMA, FDA, and CDC and (iii) a snowball search of the reference lists from the reviewed documents in the first two strategies. Additionally, BIR resources and knowledge were exchanged with other subject matter experts or task forces within trade associations including International Federation of Pharmaceutical Manufacturers and Associations (IFPMA) and Vaccines Europe – a specialized group of European Federation of Pharmaceutical Industries and Associations (EFPIA) (Bauchau et al., 2023). As the targeted literature component focused on identifying key BIR publications and datasets that were actively utilized by the MAHs, they were determined through the approaches outlined above. A formal systematic literature review was beyond the scope of this work; this is acknowledged in the limitations section.

Data were extracted on characteristics of the key initiatives from their reports, publications, dashboard or web apps, where available. These initiatives were selected based on their collaborative efforts to produce general population-based incidence rates across diverse geographic regions or countries, while establishing the infrastructure to integrate multiple data sources including health insurance, general practice, hospital, and registry data. For each initiative and where available, study characteristics (study countries, observation period, type of data sources, study population, number of AESI, etc.), information describing challenges, limitations, and recommendations for future practice were summarized.

### Consensus approach to challenges and recommendations for best practices

This working group followed the nominal group technique (Harvey and Holmes, 2012) using structured group discussions over a period of 10 months (September 2022 - June 2023) to aid contribution and agreement on factors considered critical to best practices in generation and utilization of BIRs. The expert-based judgments were collated into preliminary sets of challenges and priorities, which were then presented for critical review at the inaugural BeCOME meeting held in June 2023 in Annecy, France (Bauchau et al., 2025). This meeting convened a diverse group of international experts representing a broad spectrum of stakeholders, including representatives from industry, academia, public health and research institutes, supra-national organizations, non-government organizations, and regulatory agencies. Following discussions and documented recommendations from this multi-stakeholder workshop, the final findings were organized into the following four domains:

1. Summary of key initiatives that generated BIRs during the COVID-19 pandemic
2. Challenges encountered during the COVID-19 vaccination campaign
3. Implications of BIR on O/E analyses
4. Recommendations for best practices: Practical Guide

## Results

### Summary of key initiatives that generated BIRs during the COVID-19 pandemic

The BIR working group identified five key initiatives: ACCESS (Willame et al., 2023; Willame et al., 2021b), GVDN (Phillips et al., 2023), OHDSI (Li et al., 2021; Voss et al., 2023), BEST (Moll et al., 2023; Tworkoski et al., 2021), and Ontario studies (Nasreen et al., 2021; Nasreen et al., 2022). These initiatives generated BIRs for varying numbers of AESIs ranging from 8 (first Ontario study) (Nasreen et al., 2021) to 41 (ACCESS) (Williame et al., 2023; Williame et al., 2021b), with significant overlap among them. These BIRs were widely used by vaccine manufacturers to support COVID-19 vaccine safety monitoring during the pandemic period and continue to be used up to the present for other vaccines as well. While additional BIR publications from countries such as Korea, Scotland, and Australia were identified during this period (Huh et al., 2021; Cullen et al., 2023; Pillsbury et al., 2023), they were not considered as key initiatives due to their limited applicability for the MAHs. Table 1 summarizes these initiatives. The detailed description of these initiatives, including their respective reports, dashboards or publications on BIRs for selected AESIs, is provided in Supplementary Table S1.

**TABLE 1:** Key initiatives for background incidence rate generation supporting COVID-19 vaccine safety monitoring by vaccine manufacturers (2020-2023).

Initiatives or studies	Coverage (Countries/regions)	Observation period	Data sources	No. of AESIs	Stratification factors
ACCESS (Final report v2.0 and publication (Willame et al., 2023; Willame et al., 2021b))	Europe (Italy, Spain, Denmark, the Netherlands, Germany, France^a^, UK)	2017-2020^b^	EHR (8); Claims (2)	41	age, sex, year, data source, population with underlying conditions
GVDN (Dashboard and publication (Phillips et al., 2023; Global Vaccine Data Network, 2023))	Global (Argentina, Australia, Canada, Denmark, England, Finland, France, New Zealand^c^, Scotland, Taiwan)	2015-2020	EHR (12 sites in Dashboard; 11 sites in publication)	13	age, sex, sex-age^c^, and period combination^c^, site, healthcare settings, and time periods
OHDSI (Li et al., 2021)	Global (Australia, France, Germany, Japan, Netherlands, Spain, UK, US)	2017-2019	EHR (8) and Claims (5)	15	age, age-sex, database
OHDSI (Voss et al., 2023)	Global (Belgium, Estonia, France, Germany, Japan, Netherlands, Serbia, Spain, Turkey, UK, US)	2017-2022	EHR (12); EHR + Registry (1); Claims (8); GP (5)	16	age, database
BEST (Final report and publication (Moll et al., 2023; Tworkoski et al., 2021))	US	2017-2020^d^ (final report) 2019-2020 (publication)	Claims (5 for final report; 6 for publication)	17	age, sex, race or ethnicity (Medicare), nursing home residency (Medicare), time periods
Ontario studies (Nasreen et al., 2021; Nasreen et al., 2022)	Canada	2015-2020	Linked health administrative database (1)	8 (2021 publication) 11 (2022 publication)	age, sex, and age-sex

ACCESS, the vACCine covid-19 monitoring readinESS; AESIs, adverse events of special interest; BEST, Biologics Effectiveness and Safety; COVID-19, coronavirus disease 19; EHR, electronic healthcare record; GP, general practitioner; GVDN, Global Vaccine Data Network; OHDSI, Observational Health Data Sciences and Informatics; UK, United Kingdom; US, United States.

^a^ Data from France were not included in final report (v2.0) due to administrative constraints in data release, which prevented timely data generation.

^b^ 2010-2013 for Danish registries and 2014-2017 for German Pharmacoepidemiological Research Database (GePaRD).

^c^ Data were available in Dashboard only.

^d^ 2019-2020 for Optum claims data source due to data unavailability prior to 2018 and the need for a one-year baseline period prior to study start.

ACCESS, GVDN, and OHDSI generated BIRs using different types of databases from multiple countries (primarily from Europe or North America), while BEST utilized exclusively US claims databases and Ontario studies generated BIRs from a single Canadian provincial linked health administrative database. These initiatives primarily targeted general pediatric and adult populations, with ACCESS and BEST additionally generating BIRs for specific subpopulations. For example, ACCESS covered subpopulations with underlying conditions who were at risk for developing severe COVID-19 and pregnant women (Willame et al., 2023; Willame et al., 2021b), and BEST included subpopulations such as nursing home residents and influenza vaccinated individuals (Moll et al., 2023; Tworkoski et al., 2021). Although all these initiatives generated BIRs stratified by age and sex separately, only the GVDN dashboard (Global Vaccine Data Network, 2023), OHDSI studies (Li et al., 2021; Voss et al., 2023), and Ontario studies (Nasreen et al., 2021; Nasreen et al., 2022) provided BIRs with dual stratification by both age and sex. Depending on data source and study design, these initiatives also generated BIRs stratified by other factors such as time periods (pre-COVID and peri-COVID), data source types (electronic health records [EHR], claims, registries), and/or healthcare settings (inpatients, emergency department visits, outpatients). Due to the urgent need to monitor COVID-19 vaccine safety, ACCESS and BEST released their reports in 2021 (Willame et al., 2021b; Tworkoski et al., 2021) and subsequently published their findings as manuscripts in 2022 (Willame et al., 2023; Moll et al., 2023). OHDSI and Ontario studies published their initial manuscripts in 2021 (Li et al., 2021; Nasreen et al., 2021), while GVDN published their BIRs in 2023 (Phillips et al., 2023) and simultaneously released them on their public dashboard (personal communication, May 2025).

The initiatives acknowledged several common challenges or limitations. These included the inability to validate case definitions since diagnostic codes do not always represent true disease occurrence, variable data quality and completeness across sources, and considerable variability (heterogeneity) in BIRs across populations, regions, and data sources. Some initiatives faced specific challenges related to their data sources. For example, governance issues (such as administrative constraints or lengthy governance approval process) affected French data access in the ACCESS initiative (Willame et al., 2021b), resulting in delay which was especially problematic during a pandemic response. In addition, the COVID-19 pandemic introduced specific challenges affecting BIRs, such as altered healthcare utilization patterns due to resourcing constraints, limits on elective procedures/encounters during the early pandemic months and increasing use of virtual healthcare. These factors could potentially lead to underestimation of less severe events. Overall, these challenges or limitations underscore the complexity of establishing reliable BIRs for vaccine safety monitoring during pandemic situations and emphasize the need to consider the context for generation of BIRs and careful interpretation when using these data for safety signal detection.

Additionally, the ACCESS, GVDN, OHDSI, and BEST initiatives offered high-level recommendations for generation and/or utilization of an appropriate BIR: (i) use the same database for comparing background and post-vaccination rates; (ii) select data sources with the most complete event identification; (iii) consider population characteristics when selecting appropriate BIRs; and (iv) account for temporal trends and seasonal variations when selecting BIRs.

### Challenges encountered during the COVID-19 vaccination campaign

The unprecedented scale and urgency of the COVID-19 vaccination campaign presented new levels of challenges for vaccine safety surveillance worldwide, although some similar challenges had been experienced during the 2009 H1N1 pandemic. Furthermore, the pandemic environment created multiple obstacles to effectively generate and utilize these rates for the extensive and evolving lists of AESIs. Built on the review of key initiatives that generated the BIRs during the COVID-19 pandemic, five major challenges were further identified through a combination of the BIR working group members’ professional experience and structured discussions and collaborative discussions held during the inaugural BeCOME meeting. These challenges are described below.

#### Timeliness

**FIGURE 1:**
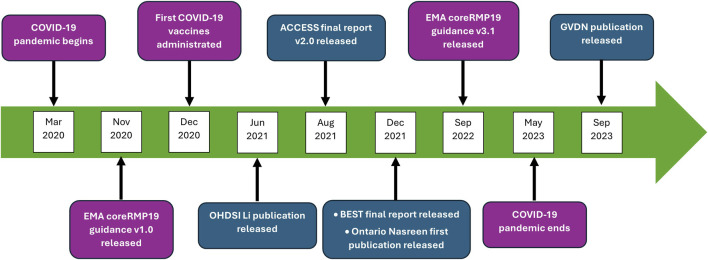
Timeline of key regulatory guidance, COVID-19 vaccine administration, and key initiatives on earliest available background incidence rates during the pandemic (2020–2023). This timeline shows the chronological relationship between pandemic milestones, first COVID-19 vaccine administration, EMA coreRMP19 guidance releases, and five key initiatives that provided earliest available background incidence rate data for COVID-19 vaccine safety monitoring. ACCESS, vACCine covid-19 monitoring readinESS; BEST, Biologics Effectiveness and Safety; COVID-19, coronavirus disease 19; EMA, European Medicines Agency; GVDN, Global Vaccine Data Network; OHDSI, Observational Health Data Sciences and Informatics; RMP, Risk Management Plan.

Timeliness refers to whether the data are collected and available at the right time for its intended use (Data Analytics and Methods Task Force EMA, 2023). Vaccines manufacturers developed COVID-19 vaccines at an unprecedented speed, making them available for mass vaccination campaigns within a year after the pandemic began (Ledford et al., 2020; Fortner and Schumacher, 2021). However, some vaccines received authorization for use earlier than others and their MAHs had regulatory obligations to rapidly initiate passive and active safety surveillance based on RWE generation. At the time of administration of the first COVID-19 vaccines in December 2020, BIR data from large scale electronic healthcare database extraction were not yet publicly available. For example, OHDSI’s publication by Li et al. (2021) became available in June 2021, and the ACCESS final report (v2.0) was released in August 2021 (Willame et al., 2021b) (Figure 1). The timing and availability of BIRs likely reflected differences in stakeholder priorities: regulatory agencies required early data for product safety monitoring, while other organizations focused on longer-term immunization program monitoring objectives.

To enable an early start and fulfilment of regulatory systematic O/E analyses requirements, MAHs, through non-competitive collaboration in the framework of the COVID Research and Development Alliance - PV and Vaccines Europe PV subgroups (COVID Research and Development Alliance, 2022; Vaccines Europe, 2020), shared experience on BIRs to achieve alignment with those obtained from published population-based epidemiological studies. As the BIR data generated from large-scale healthcare database initiatives became available over time, they were progressively incorporated as an additional source for systematic O/E analyses and other safety signal contextualization addressing evolving regulatory requirements. Specifically, the EMA requested the use of the BIRs generated from the ACCESS initiative to perform O/E analyses to be included in COVID-19 vaccine monthly summary safety reports (SSR), as outlined in core RMP19 requirements and guidance v3.1 (European Medicine Agency, 2022).

#### Heterogeneity

Heterogeneity in BIRs refers to the variability or differences in the rates of a single AESI across different populations, regions, or time periods. Among the key initiatives presented in Table 1, large variability of BIRs was observed across all multi-database studies, regardless of whether they used multiple data sources from different countries or within the same country.

**FIGURE 2:**
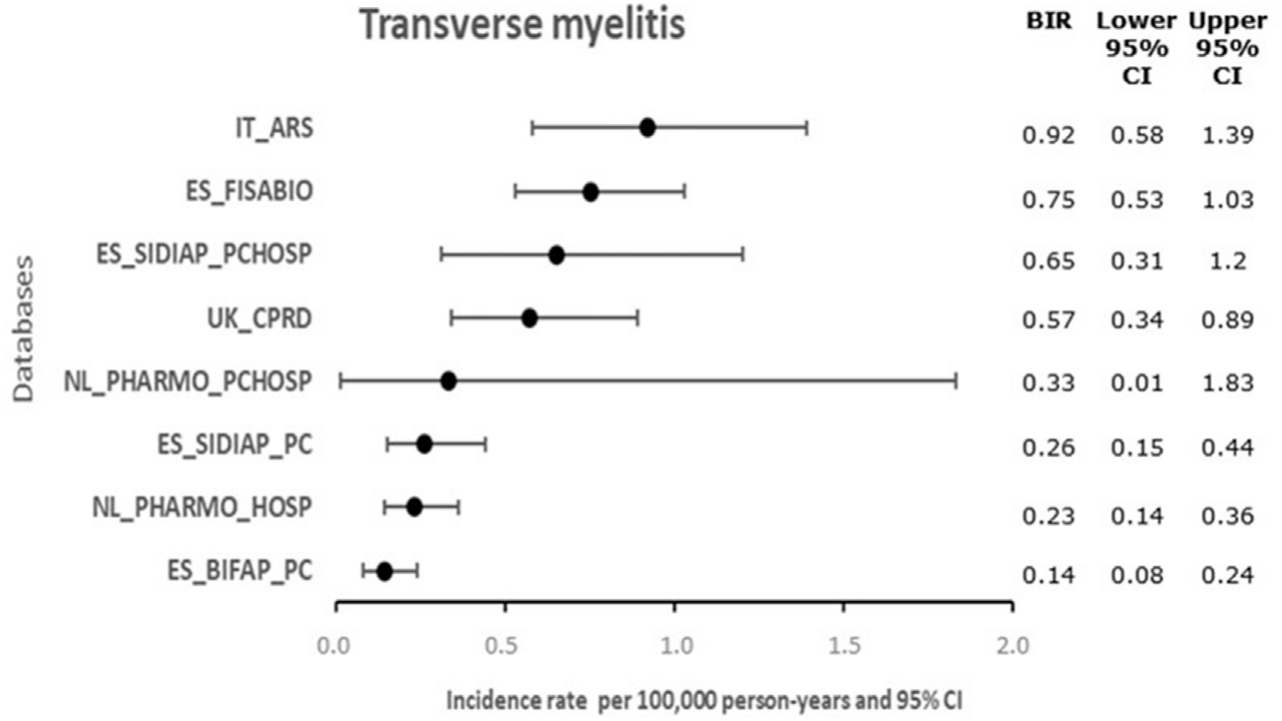
Overall background incidence rates of transverse myelitis in 2019 from ACCESS initiative using different European databases. Background incidence rates for transverse myelitis demonstrated substantial variation across data sources in ACCESS using a common data model, with rates differing by approximately 6-fold. This heterogeneity illustrates the challenges associated with selecting appropriate reference rates for observed-to-expected analyses. Data sources included in the ACCESS report: General Practitioner (GP): Base de Datos para la Investigación Farmacoepidemiológica en el Ámbito Público - Spain (ES_BIFAP_PC); Sistema d'Informació per al Desenvolupament de la Investigació en Atenció Primària - Spain (ES_SIDIAP_PC); Clinical Practice Research Datalink - UK (UK_CPRD). GP and inpatient: ES_BIFAP_PC_HOSP; PHARMO Database Network - Netherlands (NL_PHARMO_PC_HOSP). Inpatient: (NL_PHARMO_HOSP). Inpatient and Emergency room: Agenzia Regionale di Sanita’ della Toscana - Italy (IT_ARS). In and outpatient: The Valencia Health System Integrated Database - Spain (ES_FISABIO). Created based on the ACCESS report v2.0 (Willame et al., 2021b). ACCESS, vACCine covid-19 monitoringreadinESS; BIR, backgroundincidencerate; CI, confidence interval.

**FIGURE 3:**
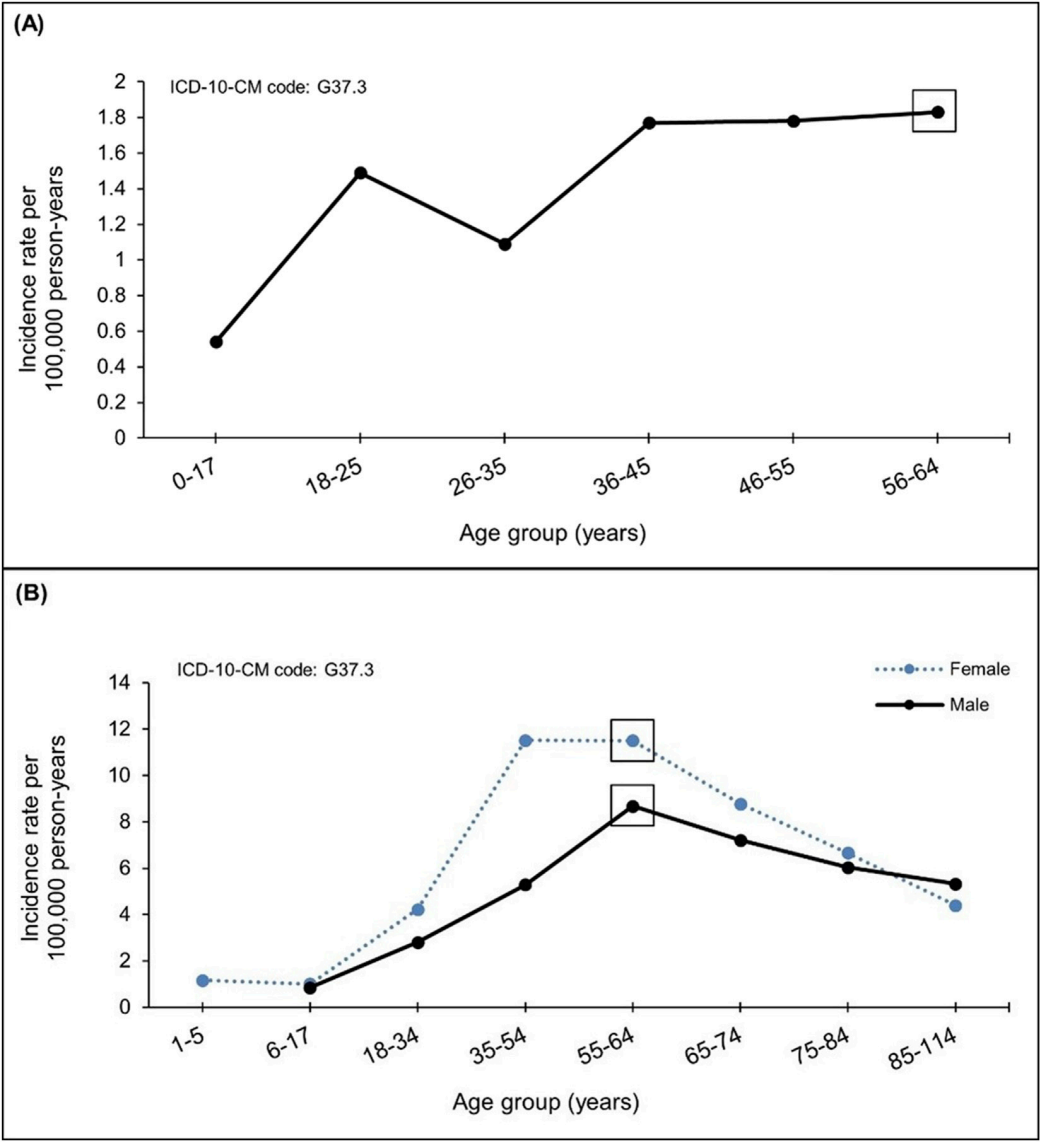
Background incidence rates of transverse myelitis from two studies using US claims databases. Background incidence rates for transverse myelitis demonstrated substantial heterogeneity between two US claims database studies, with rates differing by 4- to 6-fold. Non-standardized stratification by age/sex across studies presented challenges for selecting appropriate reference rates for observed-to-expected analyses. References (Moll et al., 2023; Li et al., 2021). **(A)** FDA BEST Optum pre-adjudicated commercial claims in 2019. **(B)** OHDSI de-identified Clinformatics Data Mart Socio-Economic Status from 2017-2019. BEST, Biologics Effectiveness and Safety; FDA, Food and Drug Administration; ICD‐10‐CM, International Classification of Diseases, 10th Revision, Clinical modification; OHDSI, Observational Health Data Sciences and Informatics; US, United States.

Despite the implementation of a common protocol, common data models, and standardized analysis methods in multi-database initiatives, substantial disparities in BIRs for the same AESIs emerged. For instance, there was a six-fold difference in the 2019 BIRs of transverse myelitis between the lowest and highest estimates across eight European databases spanning four countries in the ACCESS initiative (Willame et al., 2021b) (Figure 2). Similar variability in BIRs was observed across different initiatives, even when using similar data sources and outcome definitions within comparable observation periods. For example, when examining transverse myelitis across BEST (Moll et al., 2023) and OHDSI (Li et al., 2021) initiatives utilizing US Optum’s pre-adjudicated commercial claims and Optum’s De-identified Clinformatics Data Mart, respectively, the BIR varied considerably across age groups. In both studies, individuals aged 55–64 years exhibited the highest rates, yet the rates differed by 4 to 6-fold between the studies (Figure 3).

While the variability in results might reflect genuine differences between studied populations and offer valuable insights, heterogeneity caused by other factors (e.g., methodological differences) complicates the selection of an appropriate BIR (Table 2). Disparities can arise from different coding systems, healthcare practices, provenance of diagnosis (primary care, hospitalized care), and type of electronic healthcare database (claims, EHR), which have all been shown to impact BIR estimates (Willame et al., 2023; Ostropolets et al., 2022; Russek et al., 2023).

**TABLE 2:** Different sources of heterogeneity^*^.

Data source	Study methodology	Outcome Definitions/Phenotypes
- Healthcare setting (place of diagnosis, e.g., hospital, primary care) - Type of data (e.g., claims [open, closed], electronic health record) - Small number suppression - Operational (e.g., data lags) - Representativeness for the target country	- Anchoring (i.e., cohort entry criteria) - Clean window - Time at risk duration - Secular trends/calendar time - Choice of estimand (underlying analytical strategy)	- Differing coding systems (e.g., Read Codes, ICD codes) - Different outcome definitions - Crude vs. validated outcome definitions

ICD, international classification of diseases.

* References: (Black et al., 2021; Willame et al., 2021b; Ostropolets et al., 2022; Clothier et al., 2024).

One study published in 2022 systematically examined the influence of various parameters on BIR calculation. This study used 12 data sources for 15 AESIs related to COVID-19 vaccines as an example, and found that in addition to age and sex, study design choices such as anchoring (e.g., healthcare visit, vaccination, or arbitrary date), clean window choice (minimum interval to classify an event as incident), and time-at-risk duration or risk window (risk period for the event to occur) significantly influence BIR estimates (Ostropolets et al., 2022).

Another recent case study of BIRs for venous thromboembolism specifically assessed between-database heterogeneity using 11 healthcare databases across six European countries. This study found that databases collecting data from different segments of the healthcare system contributed most significantly to heterogeneity in BIR estimates. Moreover, the authors advise that when pooling data, careful consideration between stratified and pooled estimates is necessary for effective pharmacoepidemiological and regulatory evaluations (Russek et al., 2023).

#### Absence of consistent AESI algorithms and validation

Code-based algorithms are typically used to identify outcomes in large electronic healthcare databases. While some AESIs can be easily identified using algorithms consisting of existing simple diagnosis codes (e.g., Guillain-Barré Syndrome, myocardial infarction), others may require using or developing more complex algorithms. This is particularly true for syndromic conditions like thrombosis with thrombocytopenia syndrome (TTS) or very specific/rare adverse events such as corneal transplant rejection, where even specific diagnosis codes may not exist.

It is important to develop accurate and sometimes very complex algorithms based on available diagnostic codes, laboratory tests, drug treatments, procedures, etc. to align AESI case definitions with clinical, Brighton Collaboration, or Medical Dictionary for Regulatory Activities (MedDRA) terms. However, data on specific diagnostic tests or procedures required for accurate AESI identification may not be collected/coded in healthcare databases sometimes, or even not be generated at the healthcare provider level. For example, US claims databases often lack laboratory test results in general, while US EHR databases may have incomplete patient histories. Therefore, reconciling case definitions used for BIR calculations in healthcare databases with those employed in spontaneous reporting systems or clinical trials may pose significant methodological challenges, e.g., for O/E analyses.

Many published BIRs are “crude” rates using unvalidated code-based algorithms, potentially introducing misclassification bias. This limitation is explicitly acknowledged by all initiatives (Supplementary Table S1). In addition, when risk of outcome misclassification is expected, it is important to validate these algorithms to inform on their accuracy in identifying outcomes of interest. Medical chart extraction and review is often considered the gold standard outcome validation method, but usually represents a lengthy and resource intensive process, especially when conducting large scale BIR estimation. It may also be infeasible, especially for rare outcomes, or when none or few of the relevant medical charts are accessible (Praet et al., 2024).

Moreover, code-based algorithm performances (sensitivity and specificity) can vary significantly across database type (e.g., EHR vs. claims databases) due to differences in data elements and quality. Alternative validation methods, such as validation through database linkage, longitudinal patient profile review, or generation of a probabilistic reference standard (e.g., PheValuator) are available to circumvent some of these technical and operational limitations, especially when BIR generation is time-sensitive (Praet et al., 2024).

#### Information unavailable for specific subpopulations or geographic locations

Most publications provided BIRs only for general population and lacked estimates within specific subpopulations such as racial/ethnic subgroups, pregnant women, or populations with pre-specified co-morbidities, etc. Although all five initiatives in Table 1 included age- and/or sex-stratified BIRs, some lacked the granular data necessary for robust safety signal assessment. For instance, while the Ontario study provided more granular estimates for paediatric population (including 0-4, 5-11, 12-15 and 16–19 years) (Nasreen et al., 2022), ACCESS used a broad category of 0–19 years (Willame et al., 2023; Willame et al., 2021b) and BEST used <18 years (Moll et al., 2023; Tworkoski et al., 2021).

Furthermore, BIRs are usually not available for many low- and middle-income countries (LMICs), particularly Africa, as compared to Europe and North America. These gaps stem from two main factors. First, the compressed timelines during public health emergencies made generating comprehensive background rates across various subpopulations impractical, as it would have delayed rapid response. Second, some regions, particularly LMICs, have limited or absent infrastructure for electronic healthcare data and resources. Together, these challenges hinder effective safety signal evaluation in specific populations and LMICs. This disparity in BIR availability raises important ethical concerns about equitable safety monitoring, as populations in LMICs may receive less comprehensive post-marketing surveillance despite potentially facing different risk profiles or healthcare contexts. Addressing these disparities require targeted solutions including capacity-building initiatives, enhanced international support, and collaborative partnerships to ensure equitable vaccine safety monitoring globally.

#### Emerging AESIs or potential risks

During the COVID-19 pandemic, the rapid accumulation of new knowledge and the need for swift decision-making created a unique environment where the evolving list of AESIs due to emerging or potential risks, such as TTS and vaccine-associated enhanced disease (VAED), among others, posed another significant challenge for risk assessment.

Thrombosis with thrombocytopenia syndrome is an extremely rare, potentially life-threatening novel adverse event following adenovirus vector COVID-19 vaccination. After initial cases reported in March 2021 (Greinacher et al., 2021; Schultz et al., 2021), TTS case definitions and management guidelines were developed quickly. The Brighton Collaboration published an interim TTS case definition in May 2021 (Brighton Collaboration, 2021), with subsequent updates in November 2021 and again later in December 2023 (Schonborn et al., 2024). In parallel, the WHO published an interim guidance for clinical case management of TTS in July 2021 (World Health Organization, 2021) and updated it in July 2023 (World Health Organization, 2023). In addition to the rapidly evolving TTS case definition, availability of information in healthcare databases might be limited (lack of information on D-dimer levels, anti-PF4 antibodies, platelet counts, and timing of thrombocytopenia diagnosis) to establish an algorithm that has sufficient specificity for robust assessment of the incidence of vaccine-induced TTS. Consequently, estimating the BIR for TTS by utilization of a standardized approach across different data sources and time periods was and remains a challenge.

Vaccine-associated enhanced disease occurs when an individual who has received a vaccine develops a more severe form of the disease upon subsequent exposed to the virus, compared to unvaccinated individuals (Munoz et al., 2021). While this phenomenon was first observed with an investigational respiratory syncytial virus vaccine (Kim et al., 1969) and an inactivated measles vaccine in the 1960s (Fulginiti et al., 1967), VAED risk for COVID-19 vaccines remains theoretical (Gartlan et al., 2022). To date, no evidence of VAED from COVID-19 vaccines has been found from clinical trials or post-marketing surveillance. Recognizing the potential risk, the Brighton Collaboration formed a VAED working group of multidisciplinary experts in March 2020 (Munoz et al., 2021). The working group acknowledged significant diagnostic challenges, noting the absence of confirmatory tests for VAED, and the potential for confusion with vaccine failure as both may present with similar clinical symptoms after vaccination. Despite establishing three levels of diagnostic certainty, generating appropriate BIRs for VAED applied for any vaccines remains problematic or impossible as the case definition composition varies.

### Implications of BIRs on O/E analyses

During the COVID-19 pandemic, regulatory requirements mandated COVID-19 vaccine manufacturers to conduct O/E analyses on numerous prespecified AESIs as part of their signal detection activities (European Medicine Agency, 2022). This deviated from the traditional application of O/E analyses, which typically focuses on signal refinement for safety concerns arising from various sources (Mahaux et al., 2016). The results and interpretations of these O/E analyses were required to be included in the monthly SSR to facilitate a rapid review of the safety profile and enable swift signal identification for COVID-19 vaccines.

As a critical parameter of O/E analysis, the BIRs should closely reflect the true incidence in the target population with minimal bias. Any inaccuracies in the BIRs can substantially distort the O/E ratio and consequently affect the interpretation of safety signals. The absence of standardized methods for selecting a suitable BIR led different organizations to apply varying criteria when choosing a BIR estimate, making it difficult to compare O/E results across different COVID-19 vaccines. However, stakeholders may use BIRs differently in O/E analysis, depending on their objectives. For example, vaccine manufacturers may conduct safety signal assessments during clinical development or post-marketing surveillance, while regulatory agencies use BIRs to monitor the safety profile of vaccines under development or newly approved vaccine products for regulatory decisions.

The selection of an appropriate BIR was further complicated by the heterogeneity in the estimates, as described above. EMA strongly recommended using ACCESS-derived BIRs in Europe (European Medicine Agency, 2022), but significant inter-source variability was observed, as illustrated in Figure 2. This variability introduced uncertainties in calculating the number of expected AESI cases, which consequently affected the O/E ratio. For instance, assuming 5 million COVID-19 vaccine doses administered and a fixed 42-day risk window for transverse myelitis, the minimum number of observed cases needed for a statistically significant O/E ratio varies substantially based on the selected ACCESS BIR: 4-6 reported cases when using lower BIRs (0.14-0.33 per 100,000 person-years) *versus* 8-11 cases with higher BIRs (0.57-0.92 per 100,000 person-years). This variability could lead to biased O/E estimates and incorrect safety signal assessments. Therefore, multiple sensitivity analyses across a range of BIR estimates were needed to address these uncertainties.

The availability of granular-level BIRs (e.g., age- and sex-specific BIRs) was also essential. For example, with myocarditis, one of the prespecified AESIs under investigation in the O/E analysis requested by regulators, age- and sex-stratified analyses proved particularly important. While overall O/E analyses indicated no elevated ratios, age-and sex-specific analyses revealed statistically significant O/E ratios within the younger age group, predominantly in men following the second dose of mRNA COVID-19 vaccines (European Medicines Agency, 2021). This finding underscores the importance of stratified BIR and granular vaccine exposure data for effectively generating evidence while fine tuning potential safety signals.

Beyond BIR selection challenges, it should be noted that O/E analyses also face other challenges and methodological considerations when estimating the “observed” component from passive reporting systems. These challenges and proposed improvements to current analytical methods are detailed in a separate publication by the BeCOME dedicated working group on O/E analyses (Serradell et al., 2025).

### Recommendations for best practices: practical guide

Background incidence rates serve as a cornerstone of vaccine safety surveillance, providing essential context for interpreting adverse events following vaccination. By establishing a critical baseline for O/E analyses, BIRs help distinguish true safety signals from coincidental events that would have occurred regardless of vaccination. This baseline provides the context necessary for effective safety signal assessment and supports evidence-based regulatory decision-making. Therefore, generating unbiased and precise BIRs in target populations is essential.

**FIGURE 4:**
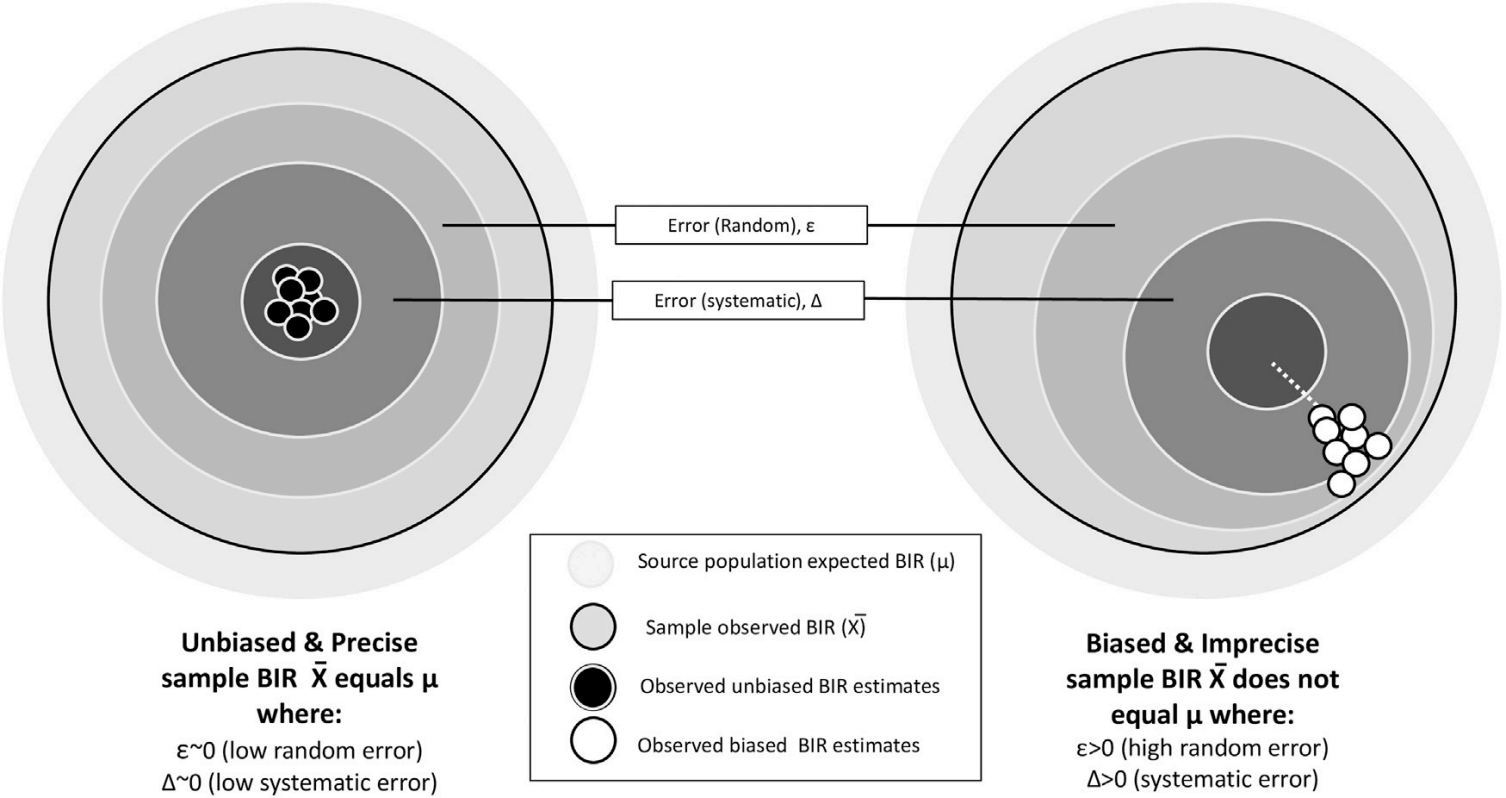
Characteristics of unbiased precise background incidence rates. To convey the idea that a sample BIR should be an unbiased estimator of the population BIR, consider the following scenario: population BIR (μ) = sample BIR (Χ) + error [random(ε) + systematic (Δ)]. A precise, unbiased BIR estimator deviates minimally from the expected population value due to negligible random and systematic error. As error magnitude increases, estimators become biased and/or imprecise. Random error sources include sampling variability, individual biological variation, and measurement inaccuracies. Systematic error sources include selection bias, poor data provenance, misclassified phenotypes, and contextual misalignment (e.g., inappropriate treatment setting or time frame). BIR, background incidence rates.

For a BIR to be unbiased and precise, the observed BIR value would have minimal uncertainty from the two fundamental sources of error: (i) random error (reduces with increasing sample size and is reflected in the width of the confidence interval) and (ii) systematic error (not affected by sample size and results in underestimation or overestimation of the true incidence) (Figure 4). Random error occurs by chance and in an unpredictable direction. On the other hand, systematic error causes BIRs to deviate from the true incidence in directions that may be predicted based on the type of systematic error.

To inform the best practices in estimating BIRs, two factors should be considered: (i) is a candidate bias certain to be present? and (ii) if present, is the candidate bias likely to introduce a small or large deviation from the true incidence? Generators and users of BIRs should therefore consider which major sources of error are likely to profoundly impact the results and which may have more inconsequential effects. For example, random error may well be the highest priority if estimating a BIR from a small database (e.g., for rare diseases), whereas if estimating a BIR using large databases, minimizing sources of systematic error may be most important. Factors such as the condition being studied, the context of care, the level of missingness, and the specific research question all play a role in determining which data source to prioritize when BIRs are heterogeneous (Clothier et al., 2024).

While random error can be reduced by increasing the sample size, when possible, systematic error requires careful consideration of many epidemiologic study design principles. Table 3 presents the BeCOME BIR working group expert opinion on a decision tool to reduce systematic error through study design that applies both in producing new BIRs from electronic healthcare databases and in assessing the appropriateness of published BIRs for a given use case. The suggested decision tool includes five methodological categories: geographic area, time period, sampling scheme, phenotype validation, and coverage of healthcare settings.

**TABLE 3:** A decision tool to guide the minimization of bias due to systematic errors in the generation and use of background incidence rates from electronic healthcare databases.

Methodological factor	Corresponding challenge	Scenario	Score	Recommendation	Example: O/E analysis for autoimmune hepatitis (AIH) (Gronbaek et al., 2020) in United States, BIR source: Grønbaek L, et al., 2020
Geographic area	- Information unavailable for certain subgroups and/or geographic regions - Heterogeneity	Region of interest is available	3	Proceed with analysis using relevant region	
		Transportable region is available (similar demographics, healthcare practices, epidemiology)	2	Proceed with analysis, noting error introduced through use of proxy population	Score: 2 – BIR study took place in England. Incidence of AIH may be similar to the US, although differences in demographics, risk factors, and healthcare practices should be considered when interpreting results
		Data unavailable from target country/region or similar	1	Do not proceed with analysis if BIR expected to be related closely to geographic factors. Otherwise, proceed noting potential error introduced through use of proxy population	
Time period	- Heterogeneity	Data reflects recent years (e.g., <5 years)	3	Proceed with analysis using most current data	
		Data is moderately recent (e.g., 5–15 years ago)	2	Proceed with analysis noting potential error introduced through changes in healthcare practices, disease trends, seasonality over time; note that older data may be prone to more substantial lack of generalizability to current period	Score: 2 – Data years in BIR source 1997-2015
		Data is relatively old (e.g., >15 years)	1		
Sampling scheme	- Heterogeneity	National database including all residents or large, representative, random sample of target population	3	Proceed with analysis using maximum data available	Score: 3 – Population-based routine healthcare records from primary, secondary, or tertiary care. Representative of England population in terms of age, sex, and ethnicity
		Large (e.g., includes many regions and/or health systems) but prone to substantial participant selection	2	Proceed with analysis noting reduced generalizability to general population; consider and report direction of expected bias	
		Small, highly selected sample (e.g., sample drawn from a single hospital)	1	Not fit-for-purpose, explore other approaches to risk contextualization	
Phenotype validation	- Absence of consistent AESI algorithms and validation - Emerging AESIs	Validated code-based algorithm compared to gold standard clinical diagnostics (e.g., chart review)	3	Proceed with analysis and report results of comparison to gold standard	
		Validated coding algorithm without clinical confirmation	2	Proceed with analysis	
		Ad-hoc phenotype algorithm designed for the current analysis	1	Proceed with analysis noting potential for misclassification; consider and report direction of expected bias	Score: 1 – Read codes and ICD-10 diagnostic codes for AIH, specific codes provided in BIR source, but no mention of validation/comparison to gold standard
Coverage of Healthcare Settings	- Heterogeneity	Complete capture of most appropriate healthcare setting(s) of diagnosis	3	Proceed with analysis	Score: 3 – BIR data source includes general practice and inpatient
		Capture of some of the most appropriate healthcare settings of diagnosis	2	Proceed with analysis noting potential underestimation of cases	
		No coverage of most appropriate diagnosis setting in selected database	1	Not fit-for-purpose, explore other approaches to risk contextualization	
Total Score	Score sum: 11 (AIH example)	**Interpretation of score** (AIH example): Proceed with O/E analysis, note potential bias due to extrapolation from England to US and measurement error from coding algorithm			

AESIs, adverse events of special interest; AIH, autoimmune hepatitis; BIR, background incidence rates; ICD-10, International Classification of Diseases, 10th version; O/E, observed-to-expected; US, United States.

Researchers are encouraged to assess the relative importance of each category when generating or using BIRs. For example, if an AESI has recently undergone a major change in diagnostic practices, time period should be considered a key priority, since it would largely bias the estimated BIR to use data from prior to the diagnostic change. To aid in assessing BIR appropriateness, we suggest assigning a quality score of 1 (low weight) through 3 (high weight) to each methodological category based on the specific scenario and recommendation. Scores can thus range from 5 to 15 (5: all scores = 1; 15: all scores = 3). Higher total scores can be generally interpreted as having less bias compared to the optimal BIR in the given application. However, critical consideration of all sources of bias, the specific objective, and intended use beyond this simplified tool is necessary in downstream uses of BIRs. Table 3 includes an illustrative example assessing a published BIR for autoimmune hepatitis (AIH) (Gronbaek et al., 2020) to be used in a hypothetical O/E analysis in the US. In this example, the BIR of AIH received a total score of 11 which can be interpreted as appropriately robust to proceed with the O/E analysis, noting potential bias introduced through extrapolation of the BIR from the UK to the US and potential measurement error due to lack of validation of the coding algorithm used to define cases.

It is imperative that all factors in Table 3 be considered together and in the context of the use case. Specifically, it is possible that a published BIR may have a high total score suggestive of low risk of biases, yet there is a key consideration that deems the particular BIR not fit-for-purpose. For example, a high-quality study using recent population-based data may have produced a BIR that is substantially higher than other published rates. For applications to PV where the goal is to be conservative in order to capture safety signals, use of a high outlier BIR may not be recommended despite receiving a high-quality score.

Background incidence rates generated from systematic reviews with meta-analyses were not considered in scope for the application of Table 3. It may be preferable to use such systematic reviews as a source to identify a single primary study of high quality and use that in downstream analyses for the relevant population rather than directly applying the meta-analytic BIR, which may be heterogenous. Considering the multifactorial study design elements critically will aid in the goal of selecting and/or generating BIRs that are unbiased and precise and therefore serve as good proxies for the source population BIRs.

It should be noted that this tool may encounter feasibility challenges, particularly in LMICs where BIRs are frequently unavailable. This highlights broader ethical concerns about inequality in vaccine safety monitoring capabilities between regions. The BeCOME LMIC working group has identified challenges in resource-constrained settings, including inadequate infrastructure, insufficient trained personnel, and poor stakeholder coordination (Bauchau et al., 2025). To address these challenges, strategic priorities have been identified: securing sustainable human and financial resources for PV systems; developing context-appropriate training programs for local staff; and fostering coordination among multiple stakeholders (e.g., vaccine manufacturers, donors, non-governmental organizations, regulators, and health ministries) (Bauchau et al., 2025). While international collaborations and WHO support are crucial, they must focus on building local capacity rather than creating dependency. Implementing these strategies will enable local BIR generation and strengthen indigenous expertise, advancing equitable vaccine safety monitoring across all populations regardless of economic circumstances.

In addition to the recommendations in Table 3, BIR generation from electronic healthcare databases should adhere to established RWE study guidelines including, but not limited to, recommendations issued by the EMA, the FDA, and the CIOMS Working Group XIII (EMA, 2024; FDA, 2023; Hennessy et al., 2025). Digitalization of healthcare data has expanded over the past few decades, allowing proactive development of more granular subpopulation data during non-emergency periods. These efforts would enhance preparedness for future public health emergencies by enabling more nuanced and equitable safety signal assessment across diverse populations. Although valuable, even BIRs derived from robust, recent, population-based studies with validated case definitions have inherent limitations that must be considered in BIR utilization and interpretation, particularly when utilized for O/E analyses.

### Methodological and contextual limitations

Several limitations warrant explicit acknowledgment. First, the methodological transparency of our targeted review process may be limited, as we did not employ a systematic review with predefined search strategies and inclusion criteria. However, this approach allowed us to conduct a focused analysis of challenges that emerged during large-scale vaccination campaigns. Second, our analysis predominantly relies on key initiatives where datasets originate primarily from high-income countries, which may not adequately represent the situations in resource-constrained settings such as LMICs. Third, some initiatives discussed depend on case definitions that have not been fully validated across diverse populations and healthcare contexts, potentially affecting the accuracy and comparability of BIRs. Finally, this paper presents experiences and perspectives from industry professionals. As stated in methods, we have endeavored to provide a balanced and objective assessment throughout this work. We encourage readers to interpret our findings and recommendations within this context, recognizing the unique insights that industry experience brings to understanding the practical challenges and solutions in vaccine safety monitoring. These limitations underscore the need for continued collaboration between industry, academia, regulatory agencies, and public health organizations to ensure comprehensive and unbiased approaches to vaccine safety surveillance.

## Conclusion

The COVID-19 pandemic highlighted both the critical importance and significant challenges of generating and utilizing BIRs for vaccine safety monitoring. Through collaborative initiatives such as ACCESS, BEST, OHDSI, GVDN, and others, substantial progress was made in developing BIR resources across multiple countries and healthcare databases. However, these efforts revealed persistent challenges including timeliness constraints, heterogeneity across data sources, limited or no BIRs available for key subpopulations and populations in LMICs, inconsistent or unvalidated case definitions, and difficulties addressing emerging AESIs. These challenges directly impacted O/E analyses, potentially leading to biased signal detection or assessment.

Moving forward, we propose a coordinated action plan among key stakeholders (regulatory agencies, industry, and BIR producers) to: (i) establish sustainable mechanisms for regular, periodic BIR delivery with continuous methodological improvements (such as harmonized case definitions, validation of AESI algorithms, etc.), enhanced stratification capabilities, and coverage of new AESIs; (ii) develop consensus on best practices for BIR selection and develop robust instruments to help guide decisions on relevance of factors and how they should be handled; and (iii) secure upfront resources to ensure sustainability and pandemic preparedness. Proactive development of more granular subpopulation data and data from LMICs during non-emergency periods would significantly enhance preparedness for future public health emergencies, enabling more nuanced and equitable safety signal assessment across diverse populations. By implementing these recommendations, we can build more robust vaccine safety monitoring systems capable of responding effectively to future public health emergencies while maintaining the highest standards of PV during routine vaccination programs.
