# Supplementary material for: Background incidence rates from electronic healthcare databases for vaccine safety monitoring: review of challenges from the COVID-19 vaccination campaign and proposal for best practices
Source: Front Drug Saf Regul. 2025 Dec 1;5:1651090. doi: 10.3389/fdsfr.2025.1651090 (PMC12702953; doi:10.3389/fdsfr.2025.1651090)
Supplement: Supplementary file 1 [file Table1.docx]

**Supplementary Table 1.** Key initiatives on background incidence rates of adverse events of special interest to support COVID-19 vaccine safety monitoring by vaccine manufacturers during peri- and post-pandemic (2020-2023)

| **Report, dashboard or publication & access link** | **Published date** | **Countries or regions of study** | **Observation period &**  **type of data sources** | **Study population &**  **Number of AESIs** | **Stratification factors** | **Reported challenges, limitations, or recommendations** |
| --- | --- | --- | --- | --- | --- | --- |
| **ACCESS Initiative** funded by European Medicines Agency (EMA) (EUPAS37273), Launched in May 2020; <https://vac4eu.org/covid-19-tool/> | | | | | | |
| ACCESS Final report v2.0  <https://doi.org/10.5281/zenodo.5255870> | 2021 Aug 25 | N = 7: Italy, Spain, Denmark, The Netherlands, Germany, France ^a^, UK | 2017-2020^b^  10 European data sources^a^: health insurance data (2); hospitalization record linkage data (4); data from general practitioners (GP) record (4) | - General population (41 AESIs) - At risk population for developing severe COVID-19 - Pregnant women (11 maternal and neonatal pregnant outcomes) | By age (0-19, 20-29, 30-39, 40-49, 50-59, 60-69, 70-79, ≥80), sex, time (calendar year, month) data source; IRs in at-risk population | Challenges of using distributed data network: facilitated access for some data sources, while governance issues delayed or prevented up-to-date data provision for others  Limitations: validation of the identified AESIs could not be performed |
| Willame et al 2023  <https://doi.org/10.1016/j.vaccine.2022.11.031> | 2022 Nov 22 (Epub) | N = 7: Italy, Spain, Denmark, the Netherlands, Germany, France, UK | 2017-2020^b, c^  10 European healthcare databases: Insurance claims (2); Record linkage (5); GP/pediatric medical record (3)  - grouped by the provenance of the events databases (In-Outpatient, Inpatient only, Inpatient & EMR, GP only, GP & In-Outpatient) | - General population (41 AESIs) - Population with underlying conditions | By age (0-19, 20-29, 30-39, 40-49, 50-59, 60-69, 70-79, ≥80), sex, year (2017-2019, 2020); IRs in population with underlying conditions  Age-standardized pooled IRs according to the provenance of the events databases | Limitations: case validation could not be conducted; governance approval can be a lengthy process, especially in a pandemic situation  Recommendations on how to use the BIRs:   - Use BIRs from data banks with the most complete event identification (such as GP and hospital-based data sources). Depending on events, data sources including emergency or outpatient data preferred for some events such as cerebral venous sinus thrombosis, while GPs setting preferred for anosmia-ageusia or chilblain-like lesions - Development of metrics to measure heterogeneity in data sources and guidance to define acceptable thresholds for distributed data network studies |
| GVDN – GCoVS project funded by the CDC of the US; Launched in 2021; <https://www.globalvaccinedatanetwork.org/> | | | | | | |
| Background Rates Dashboard  <https://www.globalvaccinedatanetwork.org/Data-Dashboards/Background-Rates-Dashboards> | 2023 Sep 5 (personal communication, May, 2025) | N = 10: Argentina, Australia, Canada, Denmark, England, Finland, France, New Zealand,Scotland, Taiwan | 2015-2020  Electronic healthcare data across 12 sites | - General population – patient types included hospital inpatients, outpatients, ED patients, and primary care patients (13 AESIs) | By age group intervals (5-year [preferred], 10-year, and 20-year age groups), sex, sex-age, and period combination | Limitations: data are not coded or collected for use in health research, rarely verified against a standardised case definition, codes and coding practices may change over time; BIRs are crude rates, which may be influenced by age, gender, distribution of the population, time (such as seasons and change over time), access to and use of healthcare services, etc. |
| Phillips et al 2023  <https://doi.org/10.1016/j.vaccine.2023.08.079> | 2023 Sep 5 (Epub) | N = 9: Argentina, Australia, Canada, Denmark, England, Finland, France, Scotland, Taiwan | 2015-2020  Electronic healthcare data across 11 sites:  Linked healthcare records and population denominator (6); linked healthcare and person registries (3), linked healthcare (hospital and claims) data (1); linked healthcare and population registries (1) | - General population – patient types included hospital inpatients, and ED patients (13 AESIs) | By age (0-19, 20-39, 40-59, 60-79, ≥80), sex, site, healthcare settings (inpatients and ED), and time periods (pre-pandemic [2015-19] and pandemic [2020]) | Limitations: data restrictions at each site (including age groups and healthcare setting available, different abilities of sites to link patient and population level data); Estimates based on electronically coded data and were not chart-validated. BIR variation by season was not captured.  Recommendations: Given the variability across geographical regions, BIRs should be from the most comparable population or region, or ideally, from the same data source. BIRs varied over time and were impacted by the COVID-19 pandemic, therefore it is important to select the most appropriate BIRs according to time periods. |
| OHDSI 2014; <https://www.ohdsi.org/>  The study published by Li et al 2021 was partially funded by the UK National Institute for Health Research (NIHR), European Medicines Agency, European Health Data and Evidence Network (EHDEN), US Food and Drug Administration CBER BEST initiative, and US National Library of Medicine. EHDEN has received funding from the Innovative Medicines Initiative 2 Joint Undertaking under grant agreement No 806968. The Innovative Medicines Initiative 2 Joint Undertaking receives support from the European Union’s Horizon 2020 research and innovation programme and EFPIA.  No funding for the study published by Voss et al 2023 | | | | | | |
| Li et al 2021  <https://doi.org/10.1136/bmj.n1435>  Interactive web app:  https://data.ohdsi.org/Covid19VaccineAesiIncidenceCharacterization/ | 2021 Jun 14 (Pub; Fast Track) | N = 8: Australia, France, Germany, Japan, the Netherlands, Spain, UK, US | 2017-2019  13 databases: electronic health record (EHR) (8); administrative claims (5) | - General population (15 AESIs) | By age (1-5, 6-17, 18-34, 35-45, 55-64, 65-74, 75-84, ≥85), age-sex, database | Challenges: Considerable variability with age and sex, emphasizing the need for standardization or stratification of BIRs for vaccine surveillance  Limitations: all outcomes could have been subject to measurement error; limitations relate to the use of each database: hospital admission was not available in the primary care datasets; incomplete capture of medical events in EHR datasets; lack laboratory test results in claims data  Recommendations: the same database be used to estimate post-covid-19 vaccine and background rates for comparison in vaccine safety monitoring. |
| Voss et al 2023  <https://doi.org/10.1016/j.eclinm.2023.101932>  Interactive web app:  [shiny.ohdsi.org:1010/Covid19SubjectsAesiIncidenceRate/](http://shiny.ohdsi.org:1010/Covid19SubjectsAesiIncidenceRate/) | 2023 Apr 4 (Epub) | N = 11: Belgium, Estonia, France, Germany, Japan, the Netherlands, Serbia, Spain, Turkey, UK, US | 2017-2022 (pre-pandemic background population 2017-2019; patients with COVID 2020-2022)  26 databases: EHR (12); EHR + Registry (1); Claims (8); GP (5) | - General population (16 AESIs) | By age (0-5, 6-17, 18-34, 35-54, 55-64, 65-74, 75-84, ≥85), database | Limitations: EHR databases are subject to incomplete capture of medical events that may occur but are recorded outside the participating health system. Administrative claims databases offered potentially complete data capture but lacked some important data elements such as laboratory test results. All databases represented subsets of the population in which they originate, which poses a risk of selection bias. Most outcome definitions were used in prior studies and were reviewed by clinicians and data experts; however, they were mainly based on the presence of specific diagnostic codes and were not validated further. |
| BEST/2020/the US FDA; https://bestinitiative.org/ | | | | | | |
| Final report  <https://bestinitiative.org/wp-content/uploads/2022/01/C19-Vaccine-Safety-AESI-Background-Rate-Report-2021.pdf> | Dec 2021 | N = 1; US | 2017-2020^d^  Administrative claims data sources (5) | - General population (17 AESIs and 6 negative control events) - Specific subpopulation (pediatric population ages 0-17 years; adult population ages 18-64 years; older adult population aged ≥65 years) - Influenza vaccinated subpopulation | Annual and monthly IRs by age, sex, race/ethnicity (Medicare only), and nursing home status (Medicare only), and time periods (Pre-COVID and Peri- COVID) | Limitations: May not be generalizable to non-insured or other publicly-insured (e.g., Medicaid) populations; a diagnosis code on a medical claim may not represent the true presence of a disease; length of the baseline clean period may not appropriate for some AESIs, leading to a combination of prevalent and incident cases; changes in coding can lead to changes in calculated IRs  Recommendations: AESI background rates vary widely across subpopulations, demographic strata, and data sources, and it is crucial to take these factors into account when determining the appropriate background rate to use for surveillance activities. |
| Moll et al 2023  <https://doi.org/10.1016/j.vaccine.2022.11.003> | 2022 Nov 8 (Epub) | N = 1; US | 2019-2020  Administrative claims data sources (6) | - General population (17 AESIs and 3 negative control events) | Annual 2019 by age <18; 18-64 [18-25, 26-35, 36-45, 46-55, 56-64]; ≥65; sex; race or ethnicity (Medicare); nursing home residency (Medicare); and time periods (pre-COVID-19 and peri-COVID-19 [an initial period and a later period]); by data sources; monthly 2019-2020 IRs | Limitations: Generalizability; not all AESIs definitions had been validated, nor was literature available to determine the clean period for all AESIs; presence of a diagnosis code on a medical claim may not necessarily reflect the presence of a disease; presented comparisons were unadjusted for other variables.  Recommendations: Given that AESI background rates varied by database and demographics, and some fluctuated during the calendar year, it is critical when evaluating COVID-19 vaccine safety to compare post-vaccination AESI rates with the background rates originating from the same database, standardize demographics, and account for seasonal trends. |
| Ontario, Canada studies: Ontario, Canada/2015/Supported by the Canadian Immunization Research Network (CIRN) Provincial Collaborative Network (PCN)/the Public Health Agency of Canada and the Canadian Institutes of Health Research; & Institute for Clinical Evaluative Sciences (ICES) /funded by the Ontario Ministry of Health^e^ | | | | | | |
| Nasreen et al 2021  <https://doi.org/10.1136/bmjopen-2021-052019> | 2021 Dec 17 (Epub) | N = 1; Canada | 2015-2020  Linked health administrative database in Ontario (1) | - Hospitalizations and emergency department visits among intercensal population estimates using Statistics Canada Census (8 AESIs) | Annual IRs by age (0-19, 20-29, 30-39, 40-49, 50-59, 60-69, 70-79, ≥80), sex, age and sex dual stratification in each of five pre-pandemic years (2015-2019) and 2020; Monthly IRs for 2015-2019 | Limitations: BIRs may be higher than those reported in the literature using hospitalization data alone; did not include cases that developed during the hospital stay leading to underestimates for conditions that frequently arise after hospitalization; Imperfect validity of the diagnostic codes in administrative data may have resulted in under or overestimation; there may be some overlap in clinical presentations of the AESI that may have impacted BIR estimation; did not capture out-of-province care provided to Ontario residents; may not be generalisable to other populations/settings, including jurisdictions within Canada with different population structures, distribution of risk factors, and diagnostic and coding practices. |
| Nasreen et al 2022  <https://doi.org/10.1016/j.vaccine.2022.04.065> | 2022 April 27 (Epub) | N = 1; Canada | 2015-2020  Linked health administrative database in Ontario (1) | - Hospitalizations and emergency department visits among intercensal population estimates using Statistics Canada Census (11 AESIs) | Annual IRs by age (0-4, 5-11, 12-15, 16-19, 20-24, 25-29, 30-39, 40-49, 50-59, 60-69, 70-79, ≥80), sex, age and sex dual stratification in each of five pre-pandemic years (2015-2019) and 2020  Monthly IRs for 2015-2019 in children aged 0-11 years | Limitations: BIRs may be higher than those reported in the literature using hospitalization data alone; Imperfect validity of the diagnostic codes in administrative data may have resulted in under or overestimation; increasing use of virtual healthcare during 2020 may have led to an underestimation of BIRs for less severe AESI that do not require ED visit or hospitalization; may not be generalizable to other populations or settings because background rates are population-specific and differ by calendar time, population structure, distribution of risk factors, and healthcare systems |

ACCESS, the vACCine covid-19 monitoring readinESS; BEST, Biologics Effectiveness and Safety; CDC: Centers for Disease Control and Prevention; ED: emergency department; GCoVS, Global Covid Vaccine Safety; GVDN, Global Vaccine Data Network; OHDSI, Observational Health Data Sciences and Informatics

^a^Data from France were not included due to administrative constraints in data release, which prevented timely data generation.

^b^2010-2013 for Danish registries and 2014-2017 for German Pharmacoepidemiological Research Database (GePaRD).

^c^Incidence rates were estimated for time periods that excluded the year 2020

^d^2019-2020 for Optum claims data source due to data unavailability prior to 2018 and the need for a one-year baseline period prior to study start

^e^Ministry of Long term Care also funded ICES to support the study conducted by Nasreen et al 2021
